# Suppression of FADS1 induces ROS generation, cell cycle arrest, and apoptosis in melanocytes: implications for vitiligo

**DOI:** 10.18632/aging.102452

**Published:** 2019-12-21

**Authors:** Luyan Tang, Jian Li, Wenwen Fu, Wenyu Wu, Jinhua Xu

**Affiliations:** 1 Department of Dermatology, Huashan Hospital Affiliated to Fudan University, Shanghai 200040, China

**Keywords:** vitiligo, microarray, fatty acid desaturase 1, apoptosis, melanocyte

## Abstract

Vitiligo is a potentially serious condition characterized by loss of melanin and death of melanocytes. To identify potential therapeutic targets for vitiligo, we conducted a microarray analysis of three human vitiligo specimens and paired adjacent normal tissues. Because we found that the fatty acid desaturase 1 (FADS1) gene was downregulated in vitiligo specimens, we carried out experiments to assess its role in melanocyte replication and survival. RT-qPCR was used to verify that FADS1 expression was lower in vitiligo-affected tissues and vitiligo melanocyte PIG3V cells than in matched controls or normal human epidermal PIG1 melanocytes. In addition, CCK-8, immunofluorescence, western blot and flow cytometry assay were used to detect the proliferation and apoptosis in PIG1 cells respectively. Overexpression of FADS1 promoted proliferation of PIG3V melanocytes, while FADS1 silencing inhibited proliferation and induced cell death in PIG1 melanocytes. Increased ROS generation; induction of mitochondrial-mediated apoptosis via upregulation of Bax and active caspases 3 and 9 and downregulation of Bcl-2; and cell cycle arrest via downregulation of c-Myc and Cyclin D1 and upregulation of p21 were all enhanced after FADS1 silencing in PIG1 melanocytes. These findings implicate FADS1 downregulation in the pathogenesis of vitiligo and may open new avenues for its treatment.

## INTRODUCTION

With a worldwide prevalence of about 1%, vitiligo is an acquired, chronic skin depigmentation disorder characterized by dysfunction or death of melanocytes [1, 2]. The pigmentation of skin and hair is determined by melanin synthesis, and its reduction can have serious health effects in patients with vitiligo [3, 4]. However, the molecular mechanism underlying the disappearance of melanocytes during vitiligo remains incompletely characterized. Topical corticosteroids and calcineurin inhibitors are commonly used in the treatment of vitiligo [5]. However, these therapies have severe side effects when used for a long time [6]. As research on vitiligo continues to unveil molecular factors involved in its progression, upregulation of melanogenesis and promotion of melanocyte proliferation are primary goals of therapeutic strategies for this condition [7, 8].

We previously conducted a microarray analysis to identify differentially expressed genes (DEGs) in clinical vitiligo tissue specimens. We found that compared with adjacent normal controls, vitiligo clinical specimens exhibit significant downregulation of fatty acid desaturase 1 (FADS1), a member of the fatty acid desaturase gene family located along with FADS2 at locus 11q12-13.1 [9]. FADS1 is a rate-limiting enzyme in the conversion of essential polyunsaturated fatty acids (PUFAs; e.g., omega-6 linoleic acid and omega-3 α-linolenic acid) into long-chain PUFAs (LC-PUFAs; e.g., arachidonic acid (ARA) and eicosapentaenoic acid (EPA) [10]. These molecules contribute to various cell structures and biological processes, influencing membrane composition, signal transduction, cell division, and regulation of lipid metabolism, among others [11]. Moreover, PUFAs serve as energy sources and regulate the phospholipid composition of mitochondrial membranes [12]. FADS1 localizes to the endoplasmic reticulum and mitochondria [13]. Mitochondria are vital organelles in eukaryotic cells, generating ATP for cellular reactions and also participating in signal transduction, regulation of apoptosis, and metabolism of fatty acids [14, 15]. A recent study indicated that TRPM2, a Ca^2+^ channel sensitive to oxidative stress, is upregulated in vitiligo melanocytes and that its inhibition in normal epidermal cells prevents Ca^2+^ influx, ROS accumulation, and apoptosis induced by oxidative stress [16]. Variation in tissue-specific FADS1 and FADS2 gene expression was recently associated with vitiligo through genome-wide analysis Qi et al 2018. However, whether FADS1 dysregulation contributes to vitiligo remains uncertain. In the present study, we investigated the effects of FADS1 expression on proliferation, apoptosis, and markers of mitochondrial oxidative stress in normal and vitiligo melanocytes. Our findings may be relevant to the design of new therapeutic strategies for this condition.

## RESULTS

### Differential gene expression analysis in clinical vitiligo specimens

**Figure 1:**
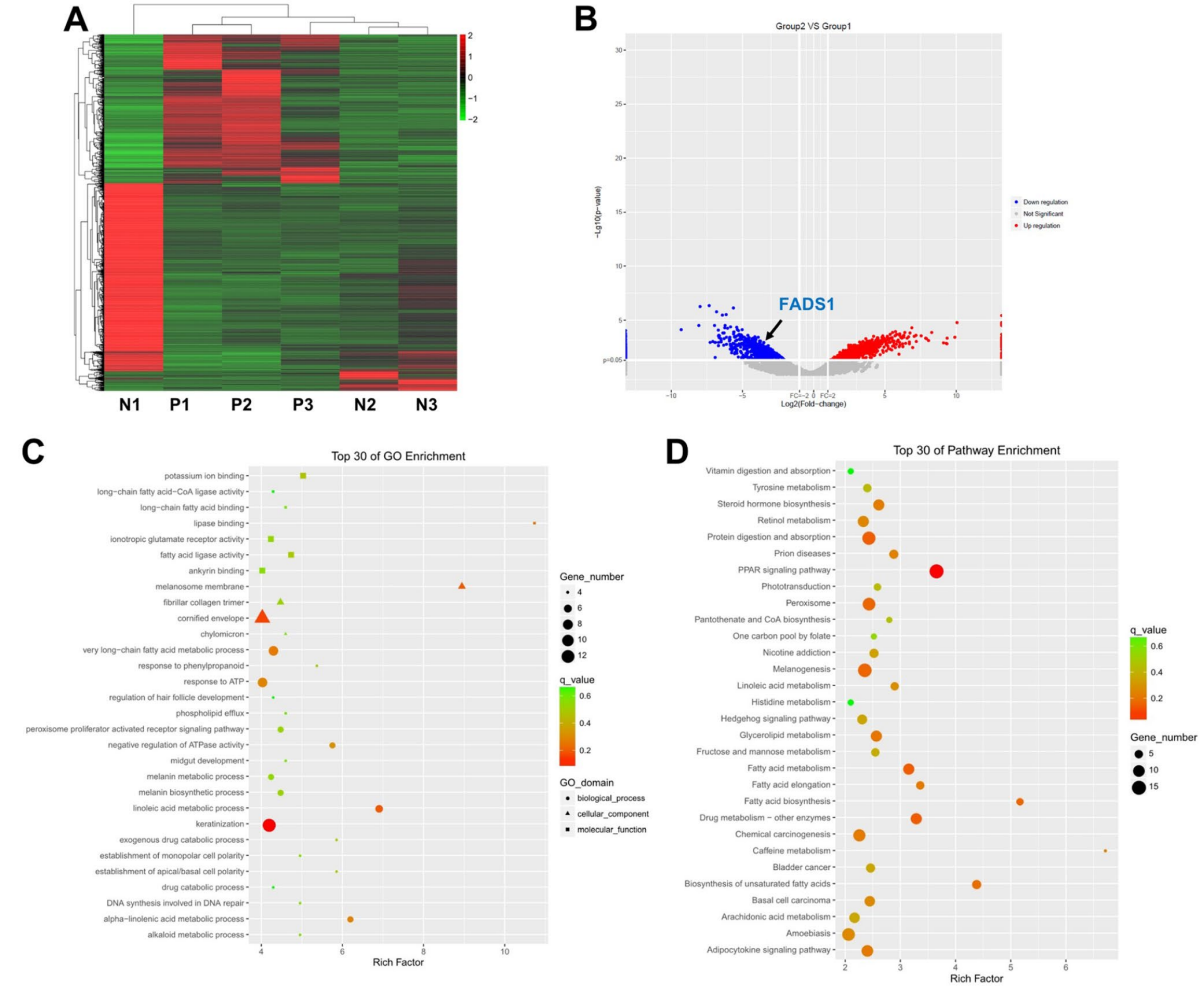
**Differential gene expression analysis in clinical vitiligo specimens.** (**A**) Heat map showing gene expression profiles in clinical samples of vitiligo and matched, adjacent normal specimens; each column represents one sample. Red and green indicate upregulation and downregulation, respectively. (**B**) Distribution of gene transcripts presented as MA plot (log2 fold-change vs log total counts); red points indicate DEGs (FADS1, log2 fold-change = -3.82, -log10 (p-value) = 2.84). (**C**) Gene ontology (GO) enrichment analysis of DEGs. (**D**) Kyoto Encyclopedia of Genes and Genomes (KEGG) analysis of DEGs. N1, N2, N3: normal specimens; P1, P2, P3: vitiligo samples.

Three lesional and non-lesional, paired epidermal samples from patients with vitiligo were collected and subjected to microarray and clustering analyses. Using a threshold of fold change (FC) ≥ 2.0 and a p-value < 0.0, the expression of 2317 genes was found to be significantly altered in vitiligo samples compared to normal controls. Specifically, 1350 genes were upregulated, and 967 genes were downregulated in vitiligo lesions as evidenced by hierarchical clustering and MA plot (Figure 1A and 1B).

Next, Gene ontology (GO) enrichment and Kyoto Encyclopedia of Genes and Genomes (KEGG) pathway analyses were used to identify the function of these DEGs. GO analysis demonstrated that most DEGs were correlated with the melanosome membrane, lipase binding, very long-chain fatty acid metabolic process, linoleic acid metabolic process, and alpha-linolenic acid metabolic process (Figure 1C). KEGG pathway analysis showed that three KEGG pathways, i.e. fatty acid metabolism, fatty acid elongation, and fatty acid biosynthesis, were related to the dysregulated DEGs (Figure 1D).

### FADS1 expression is downregulated in vitiligo melanocytes

**Figure 2:**
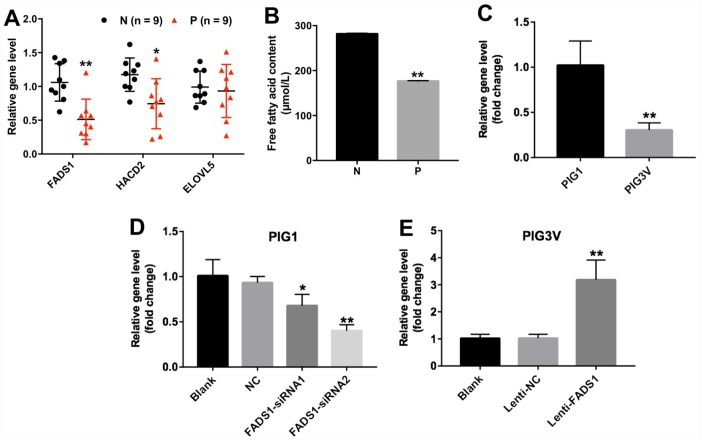
**Expression of FADS1 in human vitiligo specimens and melanocyte cell lines.** (**A**) Relative expression of FADS1, HACD2, and ELOVL5 in vitiligo samples (P) and matched normal tissues (N) by qRT-PCR (n = 9). (**B**) Free fatty acid in vitiligo samples (P) and matched normal tissues (N) detected by ELISA kit. (**C**) FADS1 expression in the vitiligo melanocyte cell line PIG3V and in the normal epidermal melanocyte cell line PIG1 detected by qRT-PCR. (**D**) FADS1 expression by qRT-PCR in PIG1 cells transfected with NC-siRNA, FADS1-siRNA1, or FADS1-siRNA2 for 72 h. (**E**) FADS1 expression by qRT-PCR in PIG3V in cells transfected with lenti-NC or lenti-FADS1 for 72 h. **P < 0.01, compared with NC cells.

Among the DEGs identified in vitiligo specimens was FADS1, which encodes a fatty acid desaturase that catalyzes the final step in the formation of EPA and ARA from PUFAs (Figure 2A). In addition, the content of free fatty acid was significantly decreased in vitiligo lesions (P = 0.01002, Figure 2B). We also used qRT-PCR to detect the expression of FADS1 in two human epidermal cell lines, namely PIG3V vitiligo melanocytes and normal PIG1 melanocytes. As shown in Figure 2C, mRNA levels of FADS1 were markedly decreased in PIG3V cells compared to PIG1 cells (P = 0.05114). These results indicated that the expression of FADS1 is downregulated in vitiligo melanocytes. To determine the function of FADS1 in melanocytes, we used two different siRNAs (FADS1-siRNA1 and FADS1-siRNA2) to knock down its expression in PIG1 cells. Through qRT-PCR, we confirmed that FADS1 mRNA level decreased by approximately 27% in PIG1 cells after transfection with FADS1-siRNA1 (P = 0.1123), whereas the level of FADS1 decreased by approximately 57% in PIG1 cells after transfection with FADS1-siRNA2 (P = 0.0025, Figure 2D). In addition, we stably overexpressed FADS1 in PIG3V cells (P = 0.0198, Figure 2E) and tested the effects of these procedures using several assays.

### Overexpression of FADS1 promotes proliferation of PIG3V vitiligo melanocytes

**Figure 3:**
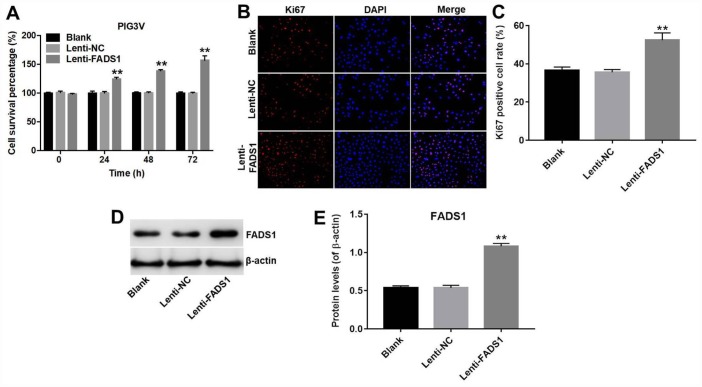
**Overexpression of FADS1 promotes proliferation of PIG3V melanocytes. PIG3V cells were transduced with lenti-NC or lenti-FADS1 for 72 h.** (**A**) Cell viability determination (CCK-8 assay). (**B**, **C**) Ki67 immunofluorescence. (**D**, **E**) Western blot detection of FADS1. **P < 0.01, compared with the NC group.

The CCK-8 assay showed that overexpression of FADS1 significantly promoted the proliferation (56% increase) of PIG3V melanocytes (P < 0.01, Figure 3A). Accordingly, in immunofluorescence assays the Ki67 positive cell rate in PIG3V cells transfected with lenti-NC or lenti-FADS1 were approximately 35% and 52%, demonstrating that upregulation of FADS1 markedly increased the number of Ki67-postive PIG3V cells compared with controls (lenti-NC transduced cells) (P = 0.0004, Figure 3B and 3C). Confirmation of FADS1 upregulation in lenti-FADS1 transduced cells was obtained by western blot (P < 0.01, Figure 3D and 3E). These data showed that FADS1 expression affects the proliferative capacity of vitiligo cells.

### Downregulation of FADS1 induces apoptosis of normal PIG1 melanocytes

**Figure 4:**
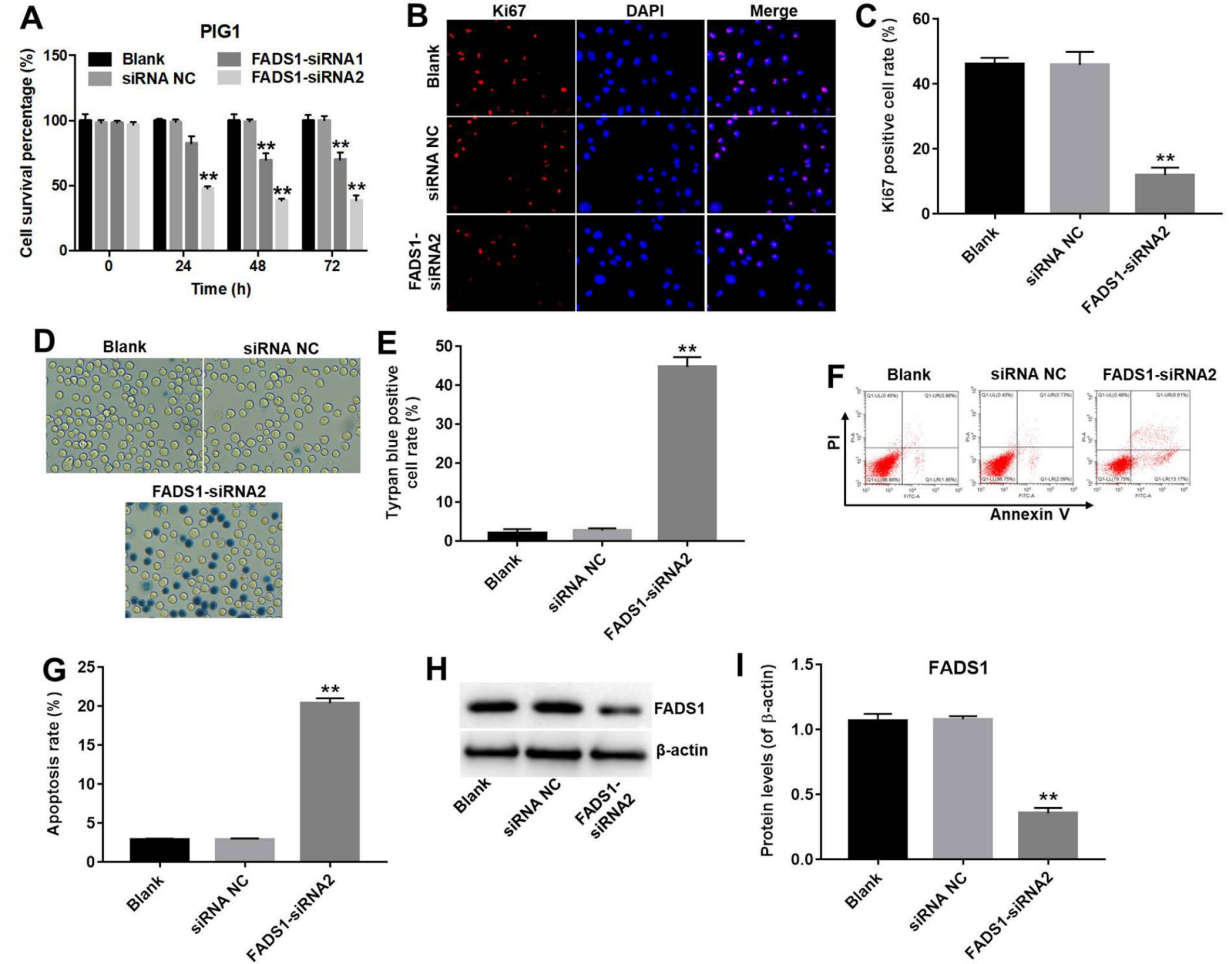
**Downregulation of FADS1 induces apoptosis in PIG1 melanocytes. PIG1 cells were transfected with NC-siRNA or FADS1-siRNA2 for 72 h.** (**A**) Cell viability determination (CCK-8 assay). (**B**, **C**) Ki67 immunofluorescence. (**D**, **E**) Cell death (trypan blue) assay. (**F**, **G**) Apoptosis assay. Annexin V/PI-stained cells were analyzed by flow cytometry. (**H**, **I**) Expression of FADS1 by western blot. **P < 0.01, compared with NC cells.

CCK-8 and Ki67 immunofluorescence assays were performed to assess the role of FADS1 on the proliferation of normal epidermal PIG1 cells. CCK-8 assay results indicated that FADS1-siRNA1 induced approximately 30% growth inhibition in PIG1 cells, whereas FADS1-siRNA2 induced approximately 61% growth inhibition in PIG1 cells (P < 0.01, Figure 4A). Therefore, FADS1-siRNA2 was used in further experiments. In addition, Ki67 immunofluorescence assay indicated that the Ki67 positive cell rate in PIG1 cells transfected with siRNA-NC or FADS1-siRNA2 were approximately 46% and 12%, indicating that downregulation of FADS1 significantly inhibited proliferation of PIG1 cells (P < 0.01, Figure 4B and 4C). Moreover, increased cell death was observed on a trypan blue staining assay (P < 0.0001, Figure 4D and 4E). The apoptosis rate in siRNA NC group was 3%, and the apoptosis rate in FADS1-siRNA2 group was 20%, demonstrating that downregulation of FADS1 markedly induced apoptosis of PIG1 cells (P < 0.01, Figure 4F and 4G). FADS1 downregulation following transfection with FADS1-siRNA2 was confirmed by western blot (P < 0.01, Figure 4H and 4I). These data showed that downregulation of FADS1 induces apoptosis in normal human PIG1 melanocytes.

### Downregulation of FADS1 decreased melanogenesis in PIG1 melanocytes

**Figure 5:**
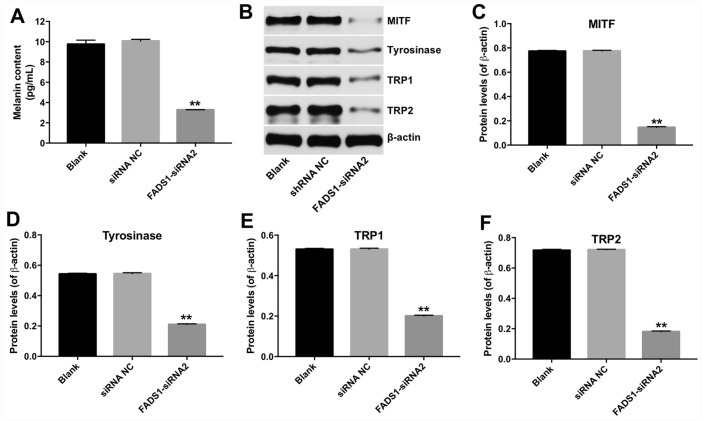
**Downregulation of FADS1 decreased melanogenesis in PIG1 melanocytes. PIG1 cells were transfected with NC-siRNA or FADS1-siRNA2 for 72 h.** (**A**) Melanin content determination (ELISA). (**B**) Expression of MITF, Tyrosinase, TRP1, and TRP2 detected by western blotting. β-actin was used as internal control. (**C**–**F**) Relative expression of MITF, Tyrosinase, TRP1, and TRP2 after normalization to β-actin. **P < 0.01, compared with NC cells.

To investigate the effect of FADS1 in PIG1 cells on melanin synthesis, melanin ELISA kit was used. As shown in Figure 5A, downregulation of FADS1 markedly decreased the content of melanin in PIG1 cells, compared with NC group. In addition, microphthalmia-associated transcription factor (MITF) is a master transcriptional regulator of melanogenic enzyme, which could regulate the transcription of three major pigmentation enzymes: tyrosinase (TYR), TRP-1, and TRP-2 [17]. Therefore, the expressions of MITF, TYR, TRP-1 and TRP-2 were detected using western blot. As indicated in Figure 5B–5F, the levels of MITF, TYR, TRP-1 and TRP-2 were significantly decreased following transfection with FADS1-siRNA2, compared with NC group. These findings suggest that downregulation of FADS1 could reduce melanogenesis via decreasing the levels of melanogenic genes.

### Downregulation of FADS1 induces ROS generation in PIG1 melanocytes

**Figure 6:**
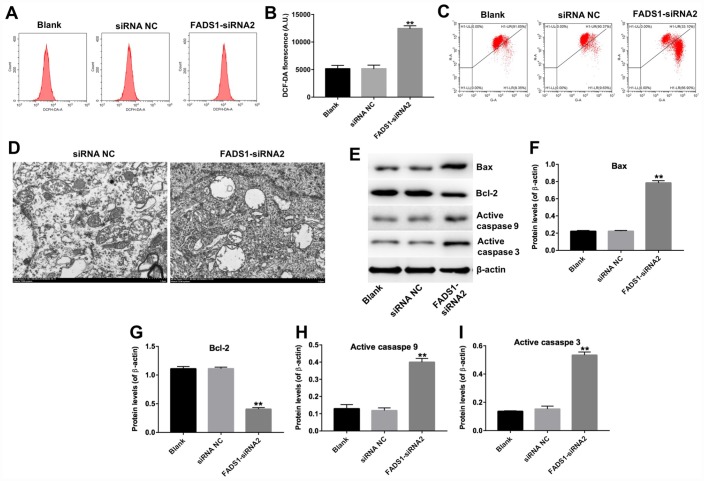
**Downregulation of FADS1 induces ROS generation, decreases MMP, and promotes expression of apoptosis markers in PIG1 cells.** PIG1 melanocytes were transfected with NC-siRNA or FADS1-siRNA2 for 24 h or 72 h. (**A**, **B**) ROS generation measured by DCF fluorescence using flow cytometry. (**C**) Changes in MMP assessed through JC-1 staining and flow cytometry. (**D**) The ultrastructural morphological changes of mitochondria in PIG1 cells observed on the TEM. (**E**) Expression of Bax, Bcl-2, active caspase 9, and active caspase 3 detected by western blotting. β-actin was used as internal control. (**F**–**I**) Relative expression of Bax, Bcl-2, active caspase 9, and active caspase 3 after normalization to β-actin. **P < 0.01, compared with NC cells.

The FADS1 protein localizes to mitochondria, the main sites of ROS production under both physiological and pathological conditions [45]. To analyze whether melanocyte death induced by FADS1-siRNA2 transfection is paralleled by enhanced ROS generation, PIG1 cells were stained with DCF-DA, a ROS indicator. As shown in Figure 6A and 6B, downregulation of FADS1 markedly increased ROS generation (P < 0.01). To further assess whether FADS1 expression affects mitochondrial function in PIG1 cells, mitochondrial membrane potential (MMP) was evaluated using the potentiometric dye JC-1. The results indicated that FADS1 silencing decreased MMP (Figure 6C). These data demonstrated that downregulation of FADS1 induces ROS generation and decreases MMP in PIG1 melanocytes. To further investigate the effect of FADS1 on the mitochondria morphology of PIG1 cells under TEM. The mitochondria of PIG1 cells in shRNA NC group exhibited complete double membrane structures, and exhibited a highly convoluted inner membrane that forms numerous invaginations called cristae (Figure 6D). However, in FADS1 siRNA2 group, the mitochondria of PIG1 cells presented swelling and distorted cristae. Moreover, the double membrane structure of mitochondria in PIG1 cells was damaged. These results indicated that downregulation of FADS1 could cause a damage to the mitochondrial morphology in PIG1 cells.

### Downregulation of FADS1 induces apoptosis of PIG1 cells through the mitochondrial-mediated apoptosis pathway

To further clarify the mechanism by which FADS1 regulates apoptosis in PIG1 cells, the expression of apoptosis-related proteins was assessed using western blot. Downregulation of FADS1 significantly upregulated the expression of Bax, active caspase 3, and active caspase 9, and markedly downregulated the expression of Bcl-2 (Figure 6E–6I). These results indicated that downregulation of FADS1 induces the mitochondrial-mediated apoptosis pathway in PIG1 cells.

### Downregulation of FADS1 inhibits cell cycle progression

**Figure 7:**
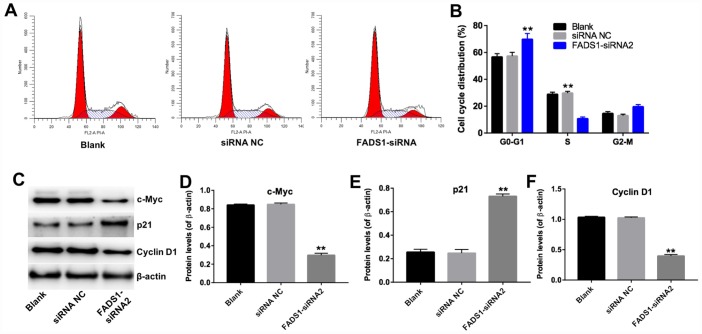
**Downregulation of FADS1 inhibits cell cycle progression in PIG1 melanocytes. PIG1 cells were transfected with NC-siRNA or FADS1-siRNA2 for 72 h.** (**A**, **B**) Cell cycle staging measured by flow cytometry. (**C**) Western blot detection of c-Myc, p21, and Cyclin D1 expression. β-actin was used as internal control. (**D**–**F**) Relative expression of c-Myc, p21, and Cyclin D1 after normalization to β-actin. **P < 0.01, compared with the NC group.

Next, the effect of FADS1 on cell cycle progression was analyzed by flow cytometry and western blotting. As shown in Figure 7A and 7B, the percentage of cells in the resting phase (G0-G1) increased to 70% following transfection with FADS1-siRNA2, while the percentage of G0-G1 cells in the siRNA NC group is 60%. In addition, the percentage of cells in the proliferative phase (S) decreased to 10% following transfection with FADS1-siRNA2, while the percentage of S phase cells in the siRNA NC group is 30%. The data showed that the percentage of cells in phases G0-G1 was significantly increased, with a concomitant reduction in the number of cells in the proliferative phase (S), in FADS1-siRNA2-transfected cells compared to NC-siRNA melanocytes. In addition, downregulation of FADS1 markedly decreased the levels of c-Myc and Cyclin D1, and increased the levels of p21 (Figure 7C–7F). These results suggest that downregulation of FADS1 inhibits cell cycle progression in PIG1 melanocytes.

### Downregulation of FADS1 inhibited melanogenesis by inhibiting the MAPK signaling pathway

**Figure 8:**
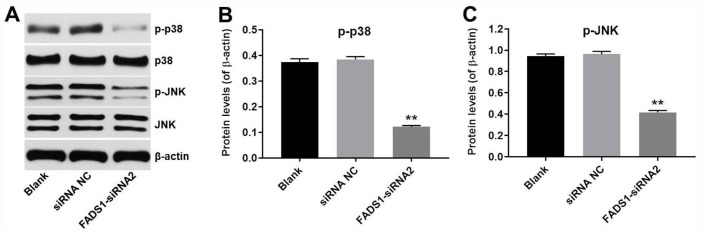
**Downregulation of FADS1 inhibited melanogenesis by inhibiting the MAPK signaling pathway.** PIG1 cells were transfected with NC-siRNA or FADS1-siRNA2 for 72 h. (**A**) Western blot detection of p-p38 and p-JNK expression. β-actin was used as internal control. (**B**, **C**) Relative expression of p-p38 and p-JNK after normalization to p38 and JNK. **P < 0.01, compared with the NC group.

To explore the involvement of MAPKs in FADS1 siRNA2-induced inhibition of melanogenesis, western blot was applied. As shown in Figure 8A–8C, downregulation of FADS1 obviously downregulated the phosphorylated levels of p38 MAPK and JNK, compared with NC group. These data indicated that downregulation of FADS1 could inhibit melanogenesis by inhibiting the MAPK signaling pathway.

## DISCUSSION

In the present study, we used microarray analysis to identify DEGs in vitiligo lesions through comparison with normal skin controls. Among the DEGs detected, we focused on FADS1, a key enzyme in the synthesis of LC-PUFAs [10]. GO and KEGG analyses confirmed the involvement of FADS1 in fatty acid metabolism, including long-chain fatty acid metabolic processing, which is essential for functional activity in mitochondria [18]. In addition, PUFA could regulate mitochondrial membrane structure and function [19]. Previous study indicated that there are significant differences in the mitochondrial morphology of vitiligo skin compared with that in health skin [20]. In addition, Dell’Anna et al. reported that alterations in energy production and mitochondrial membrane lipids could lead to melanocyte degeneration in patients with vitiligo [21]. Woo et al found that FADS1 localizes to mitochondria as well [13]. We therefore further sought to investigate whether FADS1 dysregulation impacts the progression of this condition.

We found that FADS1 was downregulated in both human vitiligo samples and vitiligo PIG3V melanocytes as to compared to matched normal skin or normal epidermal PIG1 melanocytes. Overexpression of FADS1 significantly promoted proliferation of PIG3V cells, while its downregulation reduced proliferation and induced apoptosis in PIG1 cells. These data suggest that FADS1 exerts pro-survival effects in melanocytes, and its deficiency may be critically linked to the development of vitiligo.

Several mechanisms related to mitochondrial dysfunction have been proposed to explain the degeneration of melanocytes in vitiligo, including altered expression of electron transport chain proteins and altered transmembrane distribution of cardiolipin [18]. The mitochondrial membrane potential (MMP) regulates the uncoupling effect that high fatty acid levels have on oxidative phosphorylation, and its disruption is associated with enhanced ROS generation and oxidative stress [22–24]. Studies indicate that excess ROS production contribute to loss of MMP, which in turn leads to apoptotic cell death [25–27]. Accordingly, Kang et al. recently showed that downregulation of TRPM2 can inhibit apoptosis in vitiligo melanocytes by suppressing mitochondrial ROS accumulation and MMP loss [16]. Our study shows that FADS1 downregulation in normal epidermal cells reproduces the mitochondrial deficits found in vitiligo melanocytes, namely increased ROS generation, decreased MMP, and induction of the mitochondrial-mediated apoptosis pathway.

**Figure 9:**
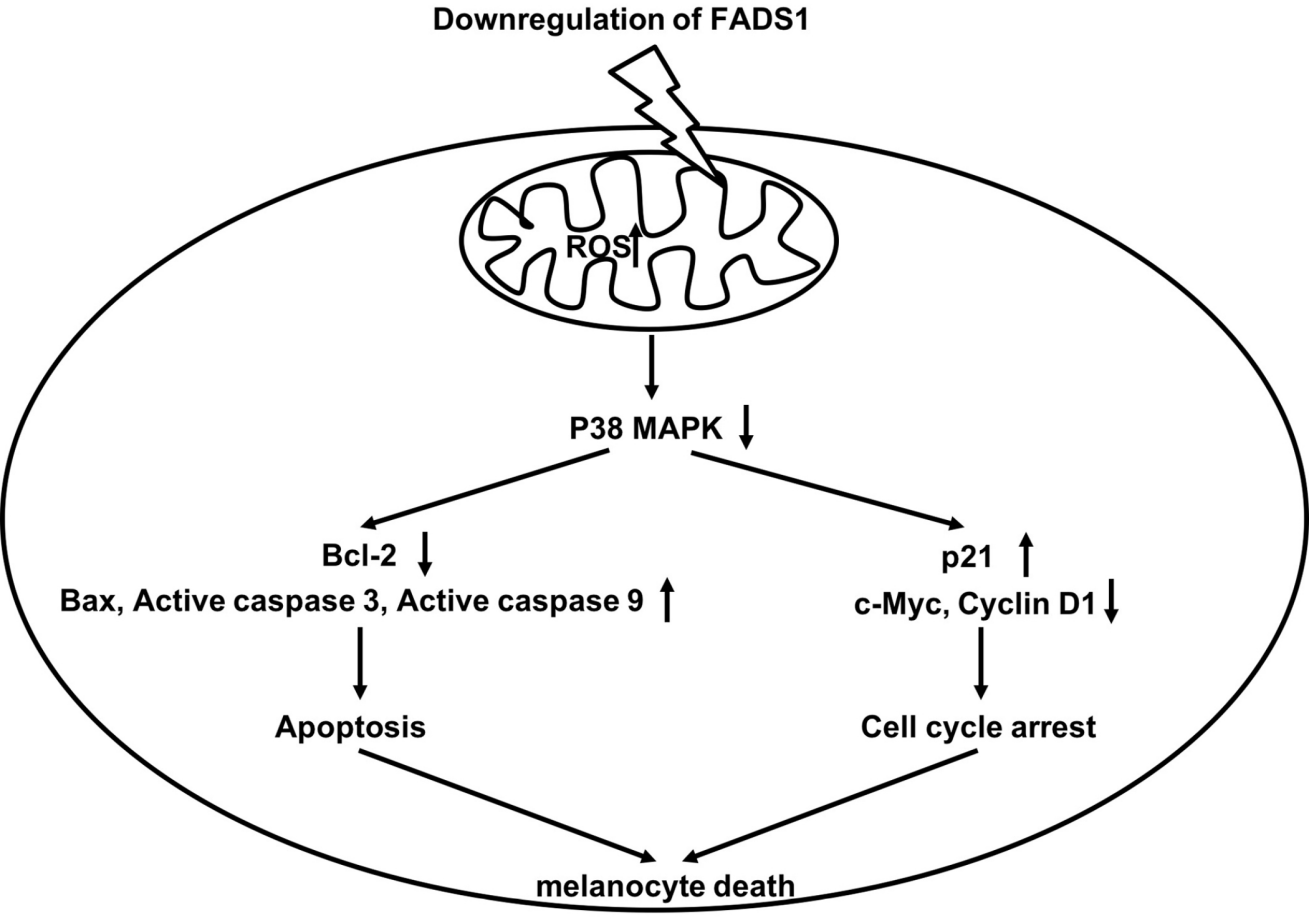
**Schematic model of apoptotic melanocyte death mediated by FADS1 downregulation. Downregulation of FADS1 increases reactive oxygen species (ROS) generation, which led to inhibition of the p38/ERK MAPK signaling pathway.** Furthermore, Downregulation of FADS1 induces apoptosis and cell cycle arrest in PIG1 cells via inhibition of the p38/ERK MAPK signaling pathway.

Loss of MMP causes the release of proteins located in the intermembrane space of the mitochondria [28], including several important apoptosis-inducing factors, such as cytochrome c, which activates caspase 9, leading in turn to caspase 3 activation [29, 30]. In addition, loss of MMP can disrupt the balance between pro-apoptotic Bax and anti-apoptotic Bcl-2 levels in mitochondria [31, 32]. Zhou et al. showed that calcitonin gene-related peptide (CGRP) together with substance P reduce melanogenesis, while CGRP exerts a dose-dependent apoptotic effect in B16F10 melanoma cells through increased expression of caspases 3 and 9 and reduced Bax/Bcl-2 ratio [33]. Our study suggests that downregulation of FADS1 in vitiligo melanocytes could be a critical determinant for apoptosis, since its suppression in normal PIG1 cells triggers apoptosis by increasing ROS generation as well as expression of Bax and active caspases 3 and 9, while downregulating Bcl-2. A schematic model of the proposed mechanism by which FADS1 downregulation leads to mitochondria-mediated apoptosis in melanocytes is shown in Figure 9.

Induction of cell cycle arrest often precedes apoptosis [34]. Zhang et al. reported that elevated ROS levels can induce cell cycle arrest through activation of the ERK signaling pathway [35]. In the present study, downregulation of FADS1 markedly inhibited cell cycle progression by inducing cell cycle arrest in G0/G1 phase. Previous studies indicate that changes in p21 and cyclin D1 levels are responsible for this effect [36]. Thus, when cells undergo DNA damage, p21 is upregulated, which inhibits expression of cyclin D1 to trigger G0/G1 arrest [37]. Our data are consistent with a mechanism by which downregulation of FADS1 in melanocytes leads to ROS generation, which in turn induces cell cycle arrest by decreasing c-Myc and Cyclin D1 levels while increasing the expression of p21 (Figure 9).

In this study, downregulation of FADS1 decreased the expressions of MITF and melanogenic genes, suggesting that downregulation of FADS1 could decrease melanogenesis in PIG1 melanocytes. JNK and p38 mitogen-activated protein kinase (MAPK) are implicated in mammalian melanogenesis [38]. MAPKs regulated melanogenesis via activation of MITF expression and the consequent increased tyrosinase expression [39]. Lee et al indicated that beauvericin could inhibit melanogenesis by suppressing the p38 MAPK signaling pathway [39]. In this study, downregulation of FADS1 inhibited phosphorylation of p38 and JNK, indicating that downregulation of FADS1 inhibited melanogenesis via inhibiting of p38 MAPK pathway (Figure 9), which was consistent with previous study. Lim et al found that naringenin induced apoptosis and ROS production in prostate cells, and decreased phosphorylation of p38 and JNK [40]. In addition, it has been shown that inhibition of MAPK signaling could suppress the proliferation, and induce the apoptosis in tumor cells [41]. Moreover, dryofragin induced apoptosis in osteosarcoma cells via inhibiting phosphorylation of p38 MAPK [42]. Collectively, FADS1-siRNA2-induced apoptosis, and cell cycle arrest in PIG1 cells was closely related to the increase in intracellular ROS, which may act as upstream factors to downregulate the p38 MAPK signaling pathway.

In summary, we observed that FADS1 is downregulated in both clinical vitiligo samples and immortalized PIG3V human vitiligo melanocytes, where forced FADS1 expression stimulates cell growth. Because FADS1 downregulation in normal melanocytes induces cell cycle arrest and cell death via ROS generation and mitochondria-mediated apoptosis, we propose that strategies aimed at restoring FADS1 expression in affected melanocytes could potentially improve therapeutic outcomes in patients with vitiligo. However, *in vivo* data are needed to validate the results of this study and confirm the role of FADS1 deficiency in vitiligo patients. In addition, the function of FADS1 on fatty acid metabolite in PIG1 cells need to be further explored.

## MATERIALS AND METHODS

### Clinical samples

A total of 60 paired vitiligo epidermal and adjacent normal specimens were obtained from the Huashan Hospital Affiliated to Fudan University, between May 2017 and July 2018. A dermatologist determined disease activity. Patients with vitiligo irrespective of gender and age were included. The exclusion criteria were: participants affected with other associated dermatoses such as psoriasis during the last 6 months, or receiving systemic corticosteroids or antioxidants. The study was approved by the Ethics Committee of Huashan Hospital Affiliated to Fudan University. Written informed consent was obtained from all participants.

### Microarray analysis

Total RNA from vitiligo specimens and adjacent normal controls was extracted with Trizol reagent (Invitrogen, Carlsbad, CA, USA) according to the manufacturer’s instructions. Differentially expressed genes (DEGs) in vitiligo samples were selected using whole-genome microarray expression profiling (Agilent Technologies, Santa Clara, CA, USA) based on the criteria of | log 2 (fold change) | >2 and adjusted P < 0.05.

### GO and KEGG pathway analyses

Gene ontology (GO) and Kyoto Encyclopedia of Genes and Genomes (KEGG) pathway analyses were used to investigate the roles of all DEGs, as previously described [43, 44]. GO analysis (http://www.geneontology.org/) was applied to elucidate genetic regulatory networks of interest by forming hierarchical categories at three levels: molecular functions, biological processes, and cellular component. KEGG pathway analyses (http://www.genome.jp/kegg/) were performed to explore significant pathways associated with the DEGs. The -log10 (p value) denotes enrichment scores that represent the significance of GO and KEGG enrichment among DEGs.

### Quantitative real-time polymerase chain reaction (qRT-PCR)

The Maxima First Strand cDNA Synthesis kit (Thermo Fisher Scientific, Waltham, MA, USA) was used to synthesize cDNA according to the manufacturer’s protocol. Then, qRT-PCR was performed using SYBR premix Ex Taq II kit (TaKaRa, Dalian, China) on a CFX96 Touch™ Deep Well Real-Time PCR Detection System (BioRad, Hercules, CA, USA) with the following primer sequences: GAPDH, forward: 5′- TCAAGAAGGTGGTGAAGCAGG -3′, reverse: 5′-TCAAAGGTGGAGGAGTGGGT -3′; FADS1, forward: 5′- TGCAATGTCCACAAGTCTGC -3′; reverse: 5′- AGCTGCCCTGACTCCTTTAG -3′. qRT-PCR reactions were performed as follows: 94°C for 10 min followed by 40 cycles of 95°C for 30 s, 60°C for 30 s, and finally, 72°C for 30 s. Relative gene expression was calculated using 2^-ΔΔCt^ method and normalized to GAPDH.

### Free fatty acid (FFA) detection content

The expression levels of FFA in vitiligo epidermal and adjacent normal specimens were assayed using the FFA assay kit (Beijing Solarbio Science and Technology Co., Ltd, Beijing, China).

### Cell culture

Two human cell lines, PIG3V (vitiligo melanocytes), and PIG1 (normal epidermal melanocytes) were purchased from American Type Culture Collection (ATCC, Rockville, MD, USA). Cells were cultured in Dulbecco’s modified Eagle medium (DMEM, Gibco, Waltham, MA, USA) supplemented with 10% fetal bovine serum (FBS, Gibco), 100 μg/L penicillin, and 100 μg/L streptomycin at 37 °C in a humidified incubator with 5% CO_2_.

### Lentivirus production and cell transduction

The lentiviral vector and the pBLLV-CMV-IRES-ZsGreen FADS1 cDNA lentiviral plasmid were purchased from GenePharma (Shanghai, China). FADS1 plasmids were co-transfected into 293T cells with pMD2.G (envelope plasmid) and psPAX2 (packaging plasmid). Lentiviral particles in the supernatant were harvested 72 h after transfection at 32°C.

PIG3V cells (4 × 10^5^ cells/well) were cultured into cell plates (60 mm) overnight at 37°C. Cells (at 50%–60% confluence) were then transduced with lentiviral vector (lenti-NC; negative control) or lentivirus FADS1 supernatants for 24 h. Then, the virus-containing media were replaced with fresh complete medium. Cell were then treated with puromycin (2.5 μg/mL, Sigma Aldrich, St. Louis, MO, USA) over 3 days to select stably transduced PIG3V cells. qRT-PCR and western blot assays were used to determine the expression of FADS1 in PIG3V cells.

### siRNA and plasmid transfection

siRNAs targeting FADS1 (FADS1-siRNA), as well as control siRNA (NC-siRNA), were purchased from Santa Cruz Biotechnology (Santa Cruz, CA, USA). PIG1 cells were transfected with 5 μL siRNA for 6 h at 37°C according to the manufacturer’s specifications. Culture media were then replaced with fresh DMEM, and cells incubated for 72 h at 37°C. qRT-PCR and western blot assays were used to determine the expression of FADS1 in PIG1 cells.

### Cell viability and cell death assays

To assess cell viability, PIG3V and PIG1 cells (2×10^4^ cells per well) were seeded into 96-well plates and incubated at 37 °C for 24 h. Cells were then transfected with lenti-FADS1 or FADS1-siRNA2 for 24, 48, or 72 h, after which 10 μL CCK-8 solution (Beyotime, Shanghai, China) was added into each well for a period of 2 h at 37°C. Absorbance (450 nm) was measured using an ELISA reader (Awareness Technology ChroMate® Microplate Reader, Ramsey, MN, USA).

For cell death assays, PIG1 cells were seeded into six-well plates and incubated overnight at 37 °C. After treatment, PIG1 cells were stained with a 0.4% trypan blue solution (Gibco, Paisley, UK) and then counted using a hemocytometer (Neubauer improved, Superior Marienfeld, Lauda-Königshofen, Germany).

### Ki67 immunofluorescence

PIG3V and PIG1 cells were plated into 24-well plates and incubated overnight at 37 °C, followed by transduction/transfection with lenti-FADS2 or FASD1-siRNA2 for 72 h. Cells were washed twice with PBS, fixed in 4% polyoxymethylene for 10 min, and incubated with primary antibodies directed against Ki67 (1:1000; ab15580; Abcam) at 4°C overnight. Subsequently, cells were incubated with a fluorescein isothiocyanate (FITC)-conjugated secondary antibody at 37°C for 1 h, counter-stained with 4’-6-diamidino-2-phenylindole (DAPI), mounted, and observed using a fluorescence microscope.

### Assay for melanin content

PIG1 cells were plated into 6-well plates and incubated overnight at 37 °C, followed by transduction/transfection with FASD1-siRNA2 for 72 h. The content of melanin in PIG1 were assayed using the melanin ELISA kit (ELK Biotechnology Co., LTD, Hubei, China).

### Western blot analysis

PIG1 cells were lysed with RIPA buffer (Beyotime, Shanghai, China), and a BCA Protein Assay Kit (Thermo Fisher Scientific) was used to measure protein concentrations in the cell lysates. Protein samples were separated by 10 % SDS-PAGE gel and transferred onto PVDF membranes (Thermo Fisher Scientific). The membranes were blocked with 5% nonfat dry milk at room temperature for 1 h and then probed at 4°C overnight with primary antibodies: anti-FADS1 (1:1000; ab126706, Abcam), anti-Bax (1:1000; ab32503), anti-Bcl-2 (1:1000; ab32124), anti-active caspase 3 (1:1000; ab2302), anti-active caspase 9 (1:1000; ab219590), anti-c-Myc (1:1000; ab320702), anti-p21 (1:1000; ab218311), anti-Cyclin D1 (1:1000; ab134175), MITF (1: 1000, ab20663), Tyrosinase (1: 1000, ab170905), TRP1 (1: 1000, ab235447), TRP2 (1: 1000, ab74703), p-p38 (1: 1000, ab47363), p38 (1: 1000, ab170099), p-JNK (1: 1000, ab76572), or JNK (1: 1000, ab208035). Then, the membranes were washed and incubated with a horseradish peroxidase (HRP) conjugated goat anti-rabbit IgG H&L secondary antibody (1:5000) for 1 h at room temperature. Blot bands were detected with Amersham ECL detection reagents (GE Healthcare, Little Chalfont, Buckinghamshire, UK) according to the manufacturer’s instructions.

### Apoptosis assay

Cell apoptosis was detected by flow cytometry using an Annexin V-FITC/PI Apoptosis detection kit (KeyGen Biotech, NanJing, China). PIG1 cells were plated onto six-well plates and incubated overnight at 37°C. Following treatments, cells were collected and washed twice with cold PBS. After resuspension in 500 μL of binding buffer, cells were incubated with 5 μL of Annexin V-FITC and 5 μl of PI at room temperature in the dark for 20 min. A flow cytometer (FACSCalibur Flow Cytometer, BD Biosciences, San Jose, CA, USA) was used to detect apoptosis.

### ROS analysis

ROS generation was evaluated by staining cells with 2′,7′-dichlorodihydrofluorescein diacetate (DCFH-DA; Sigma Aldrich) according to the manufacturer’s protocol. PIG1 cells (2×10^4^ cells per well) were seeded onto black polystyrene 96-well plates overnight at 37°C, after which FADS1-siRNA2 was added into each well and incubated for 24 h for transfection. Then, cells were incubated with 10 mM DCFH-DA for 30 min in the dark, and fluorescence detected by flow cytometry.

### Mitochondrial membrane potential assay

JC-1 staining was performed as previously described [45]. Following treatments, PIG1 cells were incubated with 2 mL JC-1 staining reagent for 20 min at 37°C in the dark. After that, cells were washed three times with PBS, and then resuspended in PBS for analysis by flow cytometry.

### Mitochondrial morphologic change

Mitochondrial morphologic changes were observed by a transmission electron microscopy (TEM, H-600IV, Hitachi Ltd., Japan). After fixation in 2.5% glutaraldehyde (GA, Sigma-Aldrich, St. Louis, MO, USA) at 4°C overnight, PIG1 cells were centrifuged at 1000 rpm for 5 min. After that, the cell pellets were fixed in 2.5% glutaraldehyde for 1 h, and refrigerated overnight, and then photos were captured using a TEM.

### Cell cycle analysis

Cell cycle analysis was performed as previously described [46]. Briefly, PIG1 cells were plated into 24-well plates overnight, followed by FASD1-siRNA2 transfection for 72 h. Cells were then fixed with 70 % ethanol and stained with PI (25 μg/mL) in fluorescence-activated cell sorting buffer for 30 min at room temperature in the dark. A flow cytometer was used to detect relative cell numbers in each cell-cycle phase (G1, S, G2, and G0).

### Statistical analysis

Data analysis was conducted with GraphPad Prism version 7 (GraphPad Software, CA, USA.). All data are presented as the mean ± SD of at least three independent experiments. Comparisons between two groups were done by Student’s t-test. P < 0.05 was considered statistically significant.
